# Signaling pathways of oxidative stress response: the potential therapeutic targets in gastric cancer

**DOI:** 10.3389/fimmu.2023.1139589

**Published:** 2023-04-18

**Authors:** Yingying Liu, Yu Shi, Ruiqin Han, Chaoge Liu, Xiaogang Qin, Pengfei Li, Renjun Gu

**Affiliations:** ^1^ School of Chinese Medicine & School of Integrated Chinese and Western Medicine, Nanjing University of Chinese Medicine, Nanjing, China; ^2^ Institute for Immunology and School of Medicine, Tsinghua University, Beijing, China; ^3^ Shanghai East Hospital, Tongji University School of Medicine, Shanghai, China; ^4^ State Key Laboratory of Medical Molecular Biology, Department of Biochemistry and Molecular Biology, Institute of Basic Medical Sciences, Chinese Academy of Medical Sciences and Peking Union Medical College, Beijing, China; ^5^ Department of Oromaxillofacial - Head and Neck Surgery, Tianjin Stomatological Hospital, School of Medicine, Nankai University, Tianjin, China; ^6^ Tianjin Key Laboratory of Oral and Maxillofacial Function Reconstruction, Tianjin, China; ^7^ Traditional Chinese Medicine Hospital of Tongzhou District, Nantong, Jiangsu, China; ^8^ Department of Clinical Laboratory, Jiangsu Province Hospital of Chinese Medicine, Affiliated Hospital of Nanjing University of Chinese Medicine, Nanjing, China

**Keywords:** gastric cancer, oxidative stress, signaling pathways, pathophysiology, therapeutic targets

## Abstract

Gastric cancer is one of the top causes of cancer-related death globally. Although novel treatment strategies have been developed, attempts to eradicate gastric cancer have been proven insufficient. Oxidative stress is continually produced and continually present in the human body. Increasing evidences show that oxidative stress contributes significantly to the development of gastric cancer, either through initiation, promotion, and progression of cancer cells or causing cell death. As a result, the purpose of this article is to review the role of oxidative stress response and the subsequent signaling pathways as well as potential oxidative stress-related therapeutic targets in gastric cancer. Understanding the pathophysiology of gastric cancer and developing new therapies for gastric cancer depends on more researches focusing on the potential contributors to oxidative stress and gastric carcinogenesis.

## 1 Introduction

Gastric cancer is the third most frequent cause of cancer-related death, and the fifth most diagnosed malignancy around the world (1). Gastric cancer is the major burden in male, accounting for 20% globally, only to lung and liver cancers (2). Anatomically, true gastric adenocarcinomas (non-cardia gastric tumors) and gastro-oesophageal junction adenocarcinomas (cardia gastric cancers) are two types of gastric cancer (3). The early stages of gastric cancer are frequently clinically unconscious, and patients are typically diagnosed at an advanced stage. The prognosis is poor once the neoplastic cells invade the muscularis propria, with the 5-year survival is almost 25% in Europe and US (4–6). With the development of economy and living standards, the prevalence of *Helicobacter pylori* (*H. pylori*) which is the key risk factor of non-cardia gastric cancer has decreased (7). Despite a consistent decrease in the rates of morbidity and mortality, more cases of gastric cancer can be seen in the future because of ageing populations (8). The disease’s late diagnosis and high mortality rate reveal a lack of knowledge regarding its etiology and pathology, as well as the absence of efficient treatments. Generally, gastric cancer is a consequence of the multifactorial interplay between host genetics, microbial factors, nutrition, and environmental milieu (9), where it is thought that oxidative stress plays a crucial role in the occurrence and development of gastric cancer.

Oxidative stress is the result of an imbalance of reactive oxygen species (ROS) production and natural antioxidant defenses, which can damage biological molecules and cells, with possible effects on the entire organism (10). Numerous studies demonstrate the tight relationship between ROS and cancer, indicating that cancer cells produced more ROS than healthy cells did (11). Increased ROS levels are thought to have an oncogenic effect, inducing DNA damage and chromosomal instability to activate proto-oncogenes and inactivate tumor suppressor genes (12, 13). Additionally, ROS also serve as signaling molecules in cancer, which affect receptor and oncogene activity, as well as alter several signaling pathways or oxidizing enzymes, facilitating tumorigenesis, angiogenesis, cellular proliferation, invasiveness, and metastasis (14). However, excessive intracellular levels of ROS may promote cell death by damaging proteins, lipid bilayers, and chromosomes. Therefore, cancer cells must fight against high level of ROS to strive for progression and develop resistance to apoptosis through antioxidant defense systems, especially at early stages (15). For this reason, both eliminating and elevating ROS production are potentially effective cancer therapies despite the fact that it is a challenging notion.

According to studies, increased levels of oxidative stress are found in individuals with gastric cancer, and this contributes to the development of gastric cancer (16). The significance of the link between oxidative stress and gastric cancer is becoming increasingly clear. This article reviews the current knowledge on the roles of oxidative stress in gastric cancer and the potential therapeutic applications of manipulating related signaling pathways in oxidative stress.

## 2 ROS production and quench

The human body continuously produces ROS which are oxygen-containing oxidants with reactive properties, represented as oxygen radicals including superoxide anions ( $O_{2}^{-}$ ), hydroxyl (HO·), alkoxyl (RO·), peroxyl (RO_2_·), and certain nonradicals either oxidizing agents and/or easily converted to radical including hydrogen peroxide (H_2_O_2_), hypochlorous acid (HOCl), singlet oxygen (^1^O_2_) and ozone (O_3_) (17). Reactive nitrogen species (RNS) are nitrogen-containing chemical species, which can damage cells via nitrosative stress. Reactive nitrogen species (RNS) include nitric oxide (·NO), nonradical compounds, peroxynitrite (ONOO^–^), nitrogen dioxide (·NO_2_) and dinitrogen trioxide (N_2_O_3_) (18) (**Table 1**). Most of these molecules are produced from oxygen in numerous metabolic processes occurring throughout the body, which primarily take place in the mitochondria, endoplasmic reticulum (ER) and peroxisomes. Approximately 2% of the oxygen consumed by the mitochondria is converted into superoxide, making it one of the largest sources of endogenous ROS (19). Peroxisomes mediate the production of ROS *via* β-oxidation of fatty acid and flavin oxidase reaction and degrading ROS *via* catalase-mediated breakdown of H_2_O_2_ (20). The ER provides an oxidizing environment, which promotes the protein folding and acts as a source of ROS (21).

**Table 1:** Formation of major oxidants.

Oxidant	Formula	Equation
Superoxide anion	O2·−	NADPH + 2O_2_ → 2O2·− + NADP^+^ + H^+^ Xanthine + 2O_2_ + NAD(P)H → Uric acid + 2O2·− + NAD(P) ^+^ + H^+^ Hypoxanthine + 2O_2_ + NAD(P)H → Xanthine + 2O2·− + NAD(P) ^+^ + H^+^
Hydrogen peroxide	H_2_O_2_	Hypoxantine + H_2_O + O_2_ → Xanthine+ H_2_O_2_ Xanthine + H_2_O + O_2_ → Uric acid+ H_2_O_2_
Hydroxyl radical	OH^−^	Fe^2+^ + H_2_O_2_ → Fe^3+^+OH^−^ + OH
Singlet oxygen	^1^O_2_	HOCl → ^1^O_2_ + H^+^ + Cl^−^
Peroxyl radicals	ROO	R + O_2_ → ROO
Hypochlorous acid	HOCl	H_2_O_2_ + Cl^−^ + H^+^→ HOCl + H_2_O
Hydroperoxyl radical	HOO·	$O_{2}^{-}$ + H_2_O ↔ HOO· + OH^−^
Nitric oxide	·NO	L-arginine + O_2_→·NO + citrulline + 2H_2_O
Nitrogen dioxide	·NO_2_	RNH2 → ·NO → $NO_{2}^{-}$ → ·NO_2_ → $NO_{3}^{-}$
Peroxynitrite anion	ONOO^−^	NO· + $O_{2}^{-}$ → ONOO^−^

Enzymatic and non-enzymatic reactions are both necessary for ROS and RNS production. The main enzymes involved in enzymatic reactions are uncoupled endothelial nitric oxide synthase (eNOS), NADPH oxidase (NOX), xanthine oxidase (XO), arachidonic acid (ARA), peroxidase, and metabolic enzymes such the cytochrome P450 system, cyclooxygenase, and lipoxygenase. The major source of ROS comes from non-enzymatic processes in the mitochondrial respiratory chain (22). Generally, ROS are by-products of biological metabolism in healthy organisms, though at lower amounts, which activate different signaling pathways to promote survival, proliferation, or resistance to oxidative stress (15). However, numerous factors, including hypoxia, ER stress, infection, inflammation, environmental toxins, nutrition, and mitochondrial respiration, all participate in the excessive ROS generation in cells.

**Figure 1:**
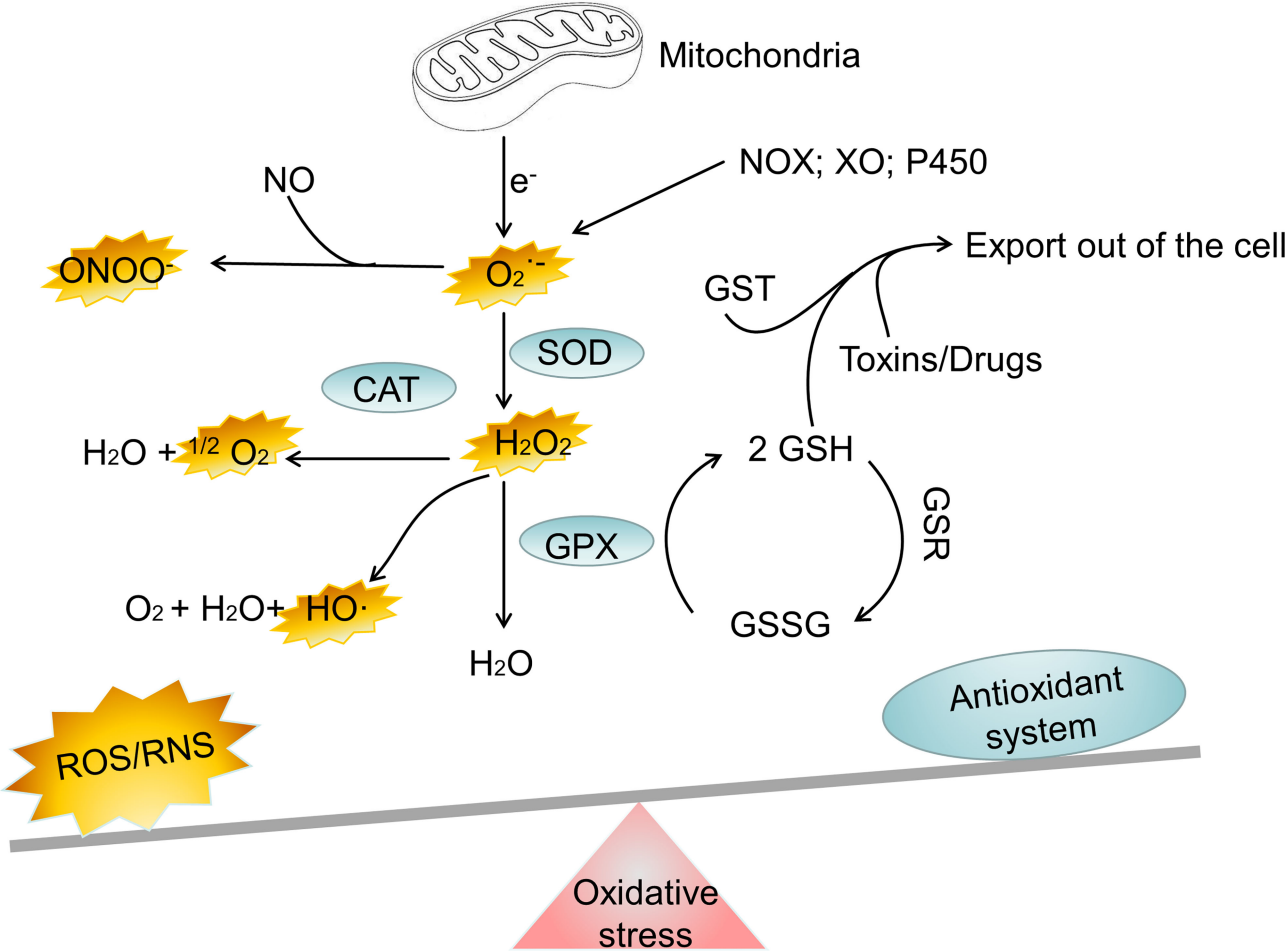
The major oxidant and antioxidant systems. NOX, NADPH oxidase; XO, xanthine oxidase; SOD, superoxide dismutase; CAT, catalase; GPX, glutathione peroxidase; GSH, glutathione; GSSG, reduced glutathione; GST, glutathione S-transferase; H_2_O_2_, hydrogen peroxide; ONOO−, peroxynitrite; HO·, hydroxyl radical; $O_{2}^{-}$ , superoxide; ^1^O_2_, singlet oxygen; Fe^2+^, Iron (II); Fe^3+^, Iron(III); ROS, reactive oxygen species; RNS, reactive nitrogen species.

Everything has two sides, and it is crucial for cell to regulate ROS levels to avoid oxidative stress. Cells have developed antioxidant defense mechanisms to scavenge ROS in maintaining homeostasis. A number of nonenzymatic and enzymatic antioxidant defense mechanisms are responsible for neutralizing ROS. The nonenzymatic defense system includes glutathione (GSH), flavonoids, dietary antioxidants such as vitamins A, C, and E, selenium and β−carotene (23). The enzymatic antioxidant system includes superoxide dismutase (SOD), glutathione peroxidase (GPX), catalase (CAT), peroxiredoxin (PRX), glutathione S-transferases (GST), glutathione reductase (GSR) and thioredoxin reductase (TRX) (24–26). It is important for cells to use these antioxidant defense mechanisms to regulate ROS levels to avoid oxidative stress. However, oxidative stress happens when the antioxidant defense system of body is overwhelmed by the production of ROS (**Figure 1**). Oxidative stress is involved in numerous human diseases, such as neurodegenerative disease, cancer, cardiovascular disease, inflammatory disease, immune system dysfunctions, allergy, diabetes, aging. For instance, inflammatory cells release chemical mediators of inflammation, particularly ROS. Due to their high reactivity, ROS typically oxidize targets with or adjacent to the intracellular compartment where they are produced, affecting surrounding neighboring cells.

## 3 Factors causing oxidative stress in gastric cancer

### 3.1 *H. pylori* and oxidative stress

A gram-negative, microaerophilic bacteria called *H. pylori* infects over 4.4 billion (or 59% of) people worldwide (7). The human gastric mucosa is selectively colonized by *H. pylori*, which can cause gastroduodenal diseases including chronic gastritis, mucosa-associated lymphoid tissue (MALT) lymphoma, peptic ulcers, and gastric adenocarcinoma (27). Sinus gastritis affects 10%-15% of *H. pylori*-infected patients and may potentially be connected to their own concurrent hypergastrinemia (28). Potential long-term complications for the patients include duodenal ulcers, intestinal metaplasia with dysplasia, gastric adenocarcinoma (non-cardia intestinal-type), and spontaneous diffuse gastric cancer (29). *H. pylori* can cause gastric lymphoma adenocarcinoma or gastric MALT lymphoma when it clings to the underlying epithelium (30, 31).

**Figure 2:**
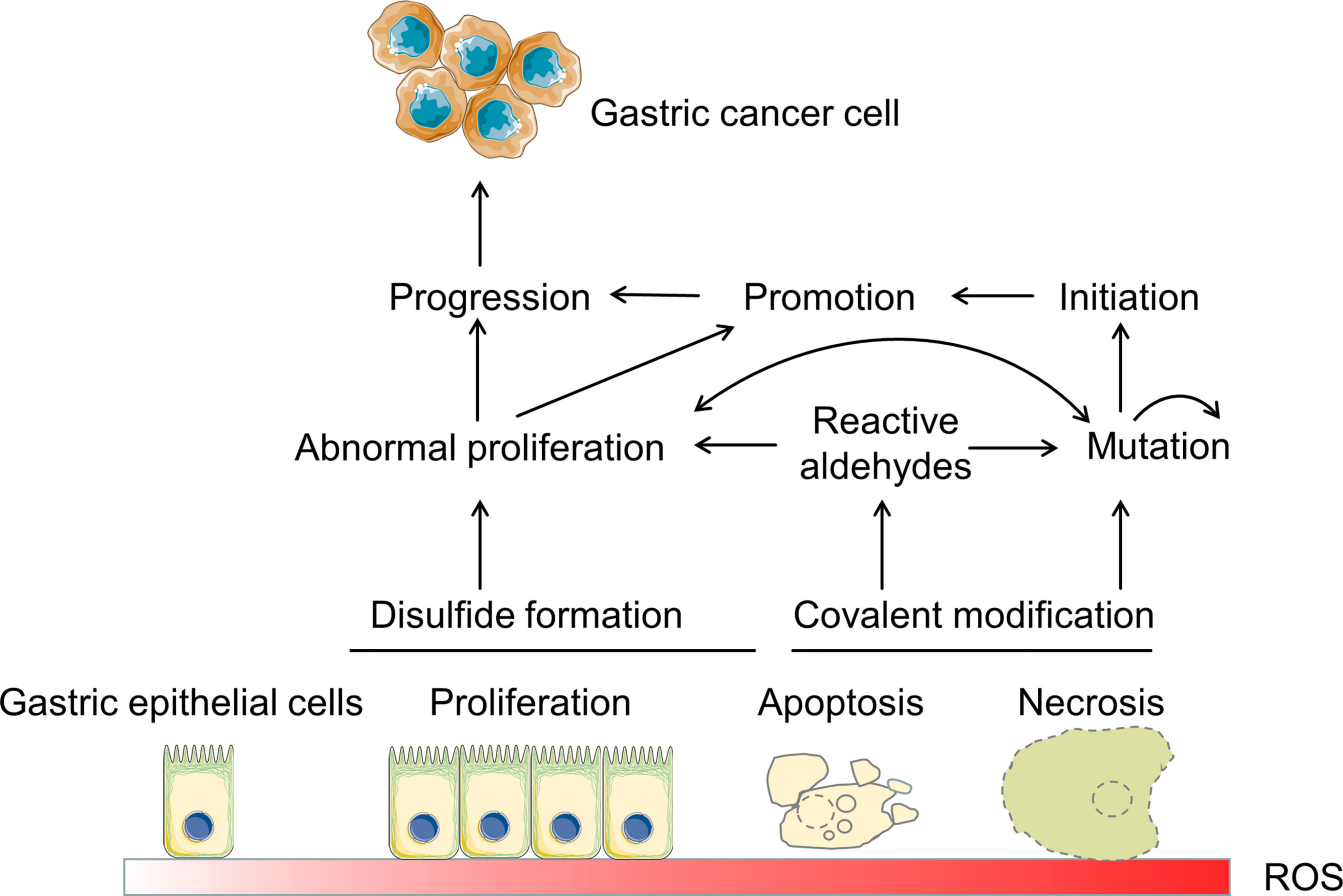
The Various pathways of ROS production and DNA damage by the epithelial and immune cells. CagA, cytotoxin-associated gene A; SMO, spermine oxidase; H_2_O_2_, hydrogen peroxide; VacA, vacuolating cytotoxin A; HO-1, heme oxygenase 1; ROS, reactive oxygen species.

The principal producers of ROS and RNS in the body are neutrophils, macrophages and gastric epithelial cells (32) (**Figure 2**). In order to kill bacteria, NOX on the neutrophil membrane catalyzes the production of ROS (33). In an effort to eradicate the infection, phagocytic cells flood the area where *H. pylori* is present. In an effort to eliminate the bacteria, both neutrophils and macrophages phagocytose produce ROS. Additionally, the inducible nitric oxide synthase (iNOS), a crucial enzyme producing Nitric oxide, is expressed in the host neutrophils and epithelial cells (34). Despite the fact that *H. pylori* activates a strong innate and adaptive response, the human immune system is typically unable to completely eliminate the infection (35). DNA damage, oxidative stress, and chronic inflammation are all directly caused by this inadequate immune response (36). Patients with *H. pylori* infections exhibit higher amounts of ROS and NO-derived metabolites, which show that iNOS has been activated (37). Compared with phagocytic cells, the epithelial cells produce ROS at a much lower, which are involved in redox-sensitive signaling pathways but may not directly eradicate *H. pylori (* 38). It is also known that the dual oxidases on the gastric epithelial cells produce H_2_O_2_ in response to infection, which likewise increases the levels of ROS (39). The environment of oxidative stress is available by the interaction of ROS generated by phagocytic and epithelial cell, which result in the growth of gastric cancer. On the one hand, one of the main causes of gastric cancer is oxidative stress by *H. pylori* infection.

The main cause is oxidative stress by *H. pylori* infection in gastric cancer. Tumor forms by *H. pylori* water extract *via* ROS production (40). Reactive oxygen metabolites are terminated by *H. pylori* treatment to eliminate the infections (41). It was feasible to ascertain the impact of bacterial eradication on oxidative stress of mucosal by contrasting the levels of nitrotyrosine and 8-hydroxy-2’-deoxyguanosine (8-oxo-dG) in antral biopsies from patients with peptic ulcer and chronic atrophic gastritis before and after eradication. Human gastric mucosa experiences less oxidative stress when *H. pylori* is removed (42). The infection of *H. pylori* can be cured by prescribed vitamins E and C with antibody therapy (43). According to recent studies, *H. pylori*-infected gastric epithelial cells produce more ROS than healthy cells do. This increased ROS production may contribute to the infection-related apoptosis (44). Furthermore, numerous virulence factors in *H. pylori* strains may lead to oxidative stress in the host. There is a higher risk of gastric carcinogenesis in patients infected with CagA-positive compared to CagA-negative strains (45). Elevated hydrogen peroxide levels and oxidative DNA damage are shown in CagA positive strains (46). IL8 and tumor necrosis factor (TNF), markers of oxidative stress and inflammation, are also increasing (47). Despite the fact that the exact mechanism by which CagA causes carcinogenesis is still unknown, it is evident that these actions can contribute to raising the chance of developing gastric cancer (48). On the other hand, gastric cells can protect themselves against oxidative stress by producing scavenger molecules.

Gastric cells can protect themselves against oxidative stress by producing scavenger molecules. Metallothioneins are important components in preventing *H. pylori*-induced gastric erosive lesions in the animal model (49). Other antioxidant systems include those that control energy metabolism globally, such as AMP-activated protein kinase (AMPK) (50) and the cytoprotective activity of nuclear factor (erythroid-derived 2)-like 2 (Nrf2) (51). At the same time, *H. Pylori* has also developed oxidative stress defense mechanisms that might encourage the acquisition of potentially cancerous traits and accelerate the development of the condition into gastric cancer (52). For example, NO levels and superoxide dismutase activity were found to have a relevant and reverse association in gastric juice of patients suffering from *H. pylori (* 53). Isogenic mutants deficient in the activities of thioredoxin (54), catalase (KatA) (55), NADPH quinone reductase (56), and superoxide dismutase (46) are sensitive to host colonization and susceptible to oxidative damage. Besides, it is interesting to note that the bacteria also produce ROS (32).

### 3.2 Smoking and oxidative stress in gastric cancer

Tobacco smoke from tar and gas phases maintain a variety of compounds, including unstable free radicals and ROS, which can harm organism through oxidative stress. The burning of tobacco produces ROS in the gas phase inhaled by smokers, as part of the mainstream smoke (57). Several rather stable free radicals in the tar phase are included in the tarry matrix, such as the quinone/hydroquinone (Q/QH2) complex (58). This Q/QH2 polymer may act as the redox system by converting pulmonary O_2_ to $O_{2}^{-}$ or additional free radicals like H_2_O_2_ and ·OH (59). Another crucial point is that, when an individual’s antioxidant defense system is weak or saturated, inhaling additional ROS or other reactive metabolites produced by the biotransformation of chemicals in tobacco smoke can increase the amount of oxidative stress caused by the gas-phase and tar-phase derived ROS (60). In addition, tar builds up in the lungs from cigarette smoke particles and processes, producing an aqueous solution that goes through redox cycling to produce different reactive species, causing damage subsequently (61).

Increasing data indicate that the release of ROS from smoking and the subsequent oxidative stress have a substantial impact on inflammation and carcinogenesis. Estimates suggest that tobacco use causes about 80,000 cases of gastric cancer annually (11% of all estimated cases) (62). Despite the decline among population-attributable fractions, smoking remained the main risk factor for men’s gastric cancer in 2012, where the incidence is substantially higher in 2020 (63). Healthy smokers may be more susceptible to oxidant-mediated tissue damage and gastric cancer because of their poor antioxidant level. The levels of thiobarbituric acid reactive substances (TBARS) are higher in smokers than in non-smokers with gastric cancer, and smokers have lower levels of SOD, CAT, GPX, GST, GSR and decreased vitamins A, E, and C (64). Low-density lipoprotein cholesterol, high-density lipoprotein cholesterol and total cholesterol all dramatically rise in non-smokers while falling in smokers, whereas these reduced in smokers (65). It has been discovered that antioxidant-rich diet significantly influenced smokers’ cellular stress protection (66). Plasma levels of malondialdehyde (MDA) were substantially higher and melatonin levels were substantially lower in smokers than non-smokers, which appears that melatonin can lessen the respiratory system damage caused by free radicals brought on by cigarette smoke (67).

## 4 Oxidative stress in gastric cancer

### 4.1 Gastric carcinogenesis

**Figure 3:**
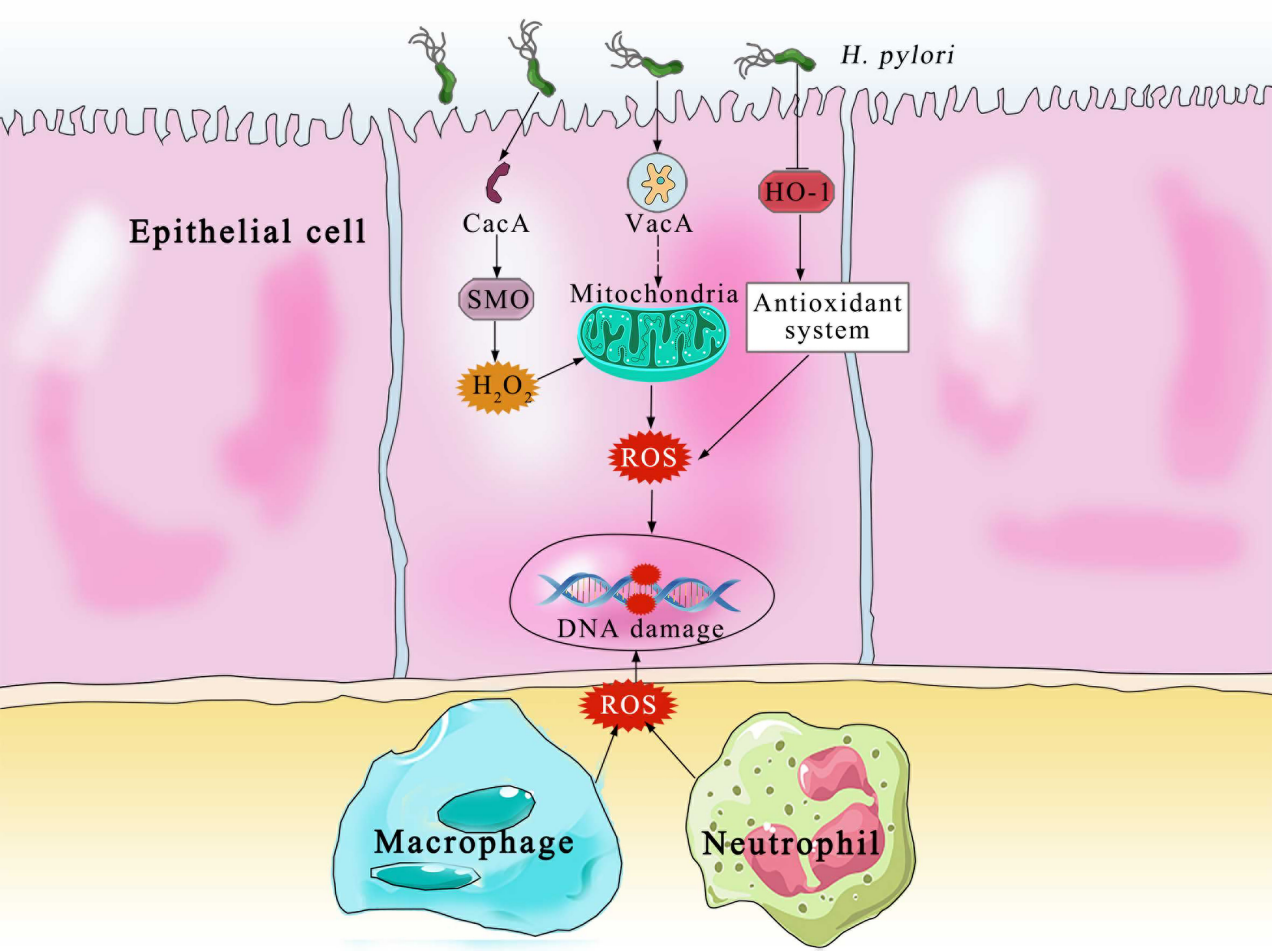
ROS and its pathophysiological effects in gastric carcinogenesis. At low to moderate concentrations, ROS function as signaling molecules that support cellular differentiation and proliferation and activate survival pathways in response to stress. Excessive ROS harms lipids, proteins, and DNA, causing mucosal injury and trigger carcinogenesis. Reactive aldehydes include 4-hydroxynonenal and other aldehydes (68). The mutator phenotype is shown by the self-directed arrow at mutation (69).

Under oxidative stress, increased ROS in cells may harm tissues and trigger carcinogenesis, especially in the gastrointestinal system (**Figure 3**). ROS are initiating factor in gastric carcinogenesis in both humans and mice. Serum and tissue samples from the human gastrointestinal have dysregulated ROS levels (41). In mice gastric cancer models induced by *H. pylori* and N-methyl-N’nitro-N’nitrosoguanidine (MNNG), the downstream pathways P53, Wnt, Ras, and mTOR are activated by ROS (70, 71). Proviral insertion in murine lymphomas 2 (PIM2) is reported to act as an oncogene in gastric cancer, controlling apoptosis *via* ROS-triggered ER stress, and promoting the development of gastric cancer (72). 13 biomarkers including β-catenin, C-MYC, GATA-4, CXCL13, DAPK1, TIMP3, DC-SIGN, EGFR, PIM2, GRIN2B, SLC5A8, VCAM-1 and CDH1 are related to the development of gastric cancer, and six of them including β-catenin, DC-SIGN, C-MYC, EGFR, CXCL13 and PIM2 have been reported overexpressed in gastric tissue from infected children and gastric cancer patients (73). Moreover, it has been shown that stomach cancer is more likely to develop as a result of the oxidative stress brought on by CagA-positive bacteria (74), in which *H pylori* CagA produces cells with oxidative DNA damage by inducing spermine oxidase (SMO), and a portion of these cells are apoptosis-resistant and therefore highly susceptible to developing cancer (75). Oxidative stress can cause DNA damage caused by *H pylori* infection. *In vitro* investigations have demonstrated that cells infected with *H pylori* that have defective DNA repair systems experience increased oxidative stress and DNA damage (76). *In vivo* studies using mice lacking a component of the base excision repair process revealed significant stomach lesions after *H pylori* infection (46). *H. pylori*’s propensity to generate DNA strand breaks undoubtedly contributes to genomic instability and may aid in carcinogenesis (77). NO can block 8-oxoguanine glycosylase from removing DNA mutations. Research has revealed that *H. pylori* infection increases phosphohistone H2AX, a marker of repair for double-strand DNA breaks (46). It has been reported that 8-hydroxy-2’deoxyguanosine buildup causes DNA damage. The loss of a base following damage would create an abasic site, which could result in a single-strand break in the DNA. Inadequate repair or continuous damage may cause double-strand breaks in the DNA, though DNA strands can be produced in various ways (46). If a cell does not heal enough fractures, it may die.

Tumor hypoxia is well recognized in oncology as a major cause of therapy resistance and poor prognosis. Hypoxia promotes the production of several gene products implicated in tumor development, invasion, and metastasis formation of gastric cancer. Hypoxia causes the production of ROS, which inhibit the degradation of the hypoxia-inducible factor 1 (HIF-1) (78). Subsequently, HIF-1α influences the expression of numerous genes that are crucial for gastric carcinogenesis. For instance, Angiogenesis is promoted by HIF-1 to stimulate the vascular endothelial growth factor (VEGF) pathway in gastric cancer (79). Caveolin-1 (Cav-1) is expressed less while induced by HIF-1, which regulates E-cadherin to cause the epithelial-mesenchymal transition (EMT) in gastric cancer (80). On the other hand, as a signaling molecule, ROS activates vital signaling pathways that are crucial to promote the onset and progression of gastric cancer. ROS, also as a second messenger, can activate tyrosine kinases and MAPK which promote cell development (81), and the protein kinase-B (Akt)/mTOR signaling pathway which promotes cell growth of gastric cancer (82). Additionally, ROS activates nuclear factor-B (NF-κB), facilitating invasion of gastric cancer (83).

Furthermore, *H. pylori*-colonized mucosal cells with deficient DNA repair systems are more vulnerable to oxidative stress and DNA damage (84). Spermine oxidase (SMOX) is activated in *H. pylori* in gastric epithelial cells, leading to oxidative stress (85). DNA damage promotes mutations of suppressor in tumor such as calcium/calmodulin dependent serine protein kinase (CASK), p53, as well as stimulation of the epidermal growth factor receptor (EFGR) signaling pathway, which are important early events in gastric carcinogenesis (86, 87). *H. pylori* colonization also negatively affects the expression of antioxidant proteins, along with epigenetic modifications and DNA methylation, such as GATA-4, GATA-5 and TWIST-1 (88), as well as miRNAs dysregulation, such as mir-21, mir-92a, mir-27a, mir-146a, mir-326, mir-155 and mir-663 (73, 89). It has been demonstrated that the expression of the purine-free/pyrimidine-free nucleic acid endonuclease 1 (APE1) is downregulated in gastric host cells infected with *H. pylori*, which ultimately reduces T-cell capacity for repair, increasing the likelihood of DNA carboxy-terminal genetic alterations. The oxidative stress defensive factors such as FOXO1, are known to be inhibited by miR-27a, which is recognized as an oncogenic miRNA in gastric cancer (90). miR-328 is downregulated in *H. pylori* -infected gastritis (90), and the low level of miR-328 activates CD44 to promote the differentiation of gastric stem cell (68). *H. pylori* increases the expression of miR-210 by controlling its methylation, which in turn suppressed dimethyl adenosine transferase 1 (DIMT1) and oncoprotein 18 or metablastic (STMN1), which promotes the proliferation of gastric epithelial cells (69). Due to the methylation of the gene promoter region by ROS, *H. pylori* infection may change the expression of miRNAs in oxidative stress, interfering with the methylation of miRNAs, which may contribute to the mechanism triggering the onset of gastric carcinogenesis.

### 4.2 Gastric adenocarcinoma and gastric cancer

The process of developing gastric cancer involves several stages, beginning with the change from normal mucosa to chronic superficial gastritis (non-atrophic gastritis). Atrophic gastritis, intestinal metaplasia, dysplasia and adenocarcinoma, among other conditions can be caused by gastritis (91). Gastritis caused by *H. pylori* is the only condition that always precedes diffuse gastric cancer. According to Correa’s idea, a series of events initiating with chronic superficial gastritis and progressing from atrophic gastritis, intestinal metaplasia, and dysplasia to gastric cancer (92). The especially high risk of cancer exists in people who have antibodies to the CagA protein, which is a marker for the more inflammatory and virulent strain of *H. pylori* that carries a pathogenicity island of genes. According to a meta-analysis of research, CagA-positive strains are two times more likely than CagA-negative strains to cause noncardia gastric cancer (93). The cag^+^ *H. pylori* strains have a stronger connection to gastric carcinogenesis than strains without cag (94). ROS or RNS production is substantially increased in vascular endothelium, gastric mucosa infected with H. pylori, and neutrophils aggregated in inflammatory mucosa (93). Following *H. pylori* infection, phagocytes that have gathered in the stomach mucosa produce O2·, HO·, and HOCl (95). Rat gastric mucosal cells have been shown to undergo apoptosis when exposed to NH_2_Cl (96).

Epstein-Barr virus (EBV) is recognized as a pathogen that causes stomach cancer. Nearly 10% of cases of gastric cancer are EBV-associated gastric cancer, which is the monoclonal proliferation of epithelial cells infected with EBV that only express a few EBV-latent genes (Latency I program) (97). The production of NH_2_Cl by infiltrating neutrophils can convert latent EBV into lytic EBV in the *H. pylori*-infected gastric, which may further contribute to gastric carcinogenesis (98). Although the function of the ROS generated by infected gastric epithelial cells is not fully known, it is thought that these ROS trigger signaling processes that control how *H. pylori* pathogenesis develops.

*H. pylori* infection directly causes oxidative stress in gastric epithelial cells by the production of ROS, and it also stimulates host responses that result in ROS and controls the production of proinflammatory cytokines, inflammation, and cell death (99). Continuous ROS production results in oncogene and tumor suppressor gene changes, as well as chromosomal abnormalities by oxidative genome damage, which includes the oxidation of guanine to form 8-OhdG and 8-oxo,7,8-dihydroguanosine (8-OHG) in RNA and DNA (100).

When compared to normal mucosa, gastric adenoma and *H. pylori*-infected or uninfected cancer tissues express ROS and APE1/Ref1 more mucosally (101). As a result of *H. pylori* infection, both the gastrointestinal lumen and gastric juice ascorbic acid content decrease. This antioxidant lessens the effects of carcinogens by lowering carcinogenic substances including nitrosamines and ROS. Depleting cellular antioxidants makes ROS more effective at killing cancer cells because this is the traditional treatment strategy for doing so. Perhaps, the disease can be regulated by blocking different antioxidant systems during neoadjuvant treatments.

### 4.3 Gastric lymphoma

Gastric MALT lymphomas are a slow-growing type of non-Hodgkins lymphoma, developed from the extranodal marginal zone of lymphoid follicles (102). Gastric MALT lymphoma is an illustration of the intimate pathogenetic relationship between chronic inflammation and tumor development. Approximately 92% of gastric MALT lymphomas have a tight connection to *H. pylori* infection which makes *H. pylori* easier to develop and diffuse (103). The *H. pylori* strains linked to gastric MALT lymphoma are less virulent than those linked to gastric adenocarcinoma. The latter strains may have the VacA m2 gene without the CagPAI, which could make *H. pylori* carriers easier to develop diffuse large B-cell lymphoma (104). *H. pylori* infection increased the incidence of low-grade gastric MALT lymphoma by an odds ratio of 2.8 times compared with *H. pylori*-negative individuals (105). Within gastric MALT lymphomas, T lymphocytes activated by *H. pylori* are responsible for B-cell proliferation (106).

Most individuals with early-stage *H. pylori* disease have been in durable remission for more than ten years after completing a single brief course of combination antibiotic therapy. A meta-analysis of more than 30 trials found that the overall remission rate of MALT lymphomas with a low histological grade that is restricted to the perigastric lymph nodes or the gastric wall (stage I or stage IIe_1_ illness) was 78% (107). Therefore, preventive removal of *H. pylori* is particularly helpful in reversing MALT lymphoma either in the early MALT stage or in the late bone marrow-involved stage. However, the recurring possibility of MALT lymphoma should not be ignored because it frequently returns several years following surgery, which may due to risk factors for gastric cancer have not been totally blocked.

Gastric MALT lymphoma is regarded as one of the greatest models for understanding how genetic events contribute to oncogenesis, influence tumor biology, govern clinical behavior, and represent feasible treatment targets. Genetic aberrations arise through the release of ROS, *H. pylori*-induced endonucleases, and other effects. Stronger oxidative stress is caused by *H. pylori* strains originating from gastric cancer in the host, and these strains may have suppressive effects on the host’s GSH-related defensive mechanisms (108). Surprisingly, the nucleotide-binding oligomerization domain protein 2 (NOD2) functions as a receptor for pattern recognition. *H. pylori* activates NF-κB signaling *via* NOD2. However, the NF-κB signaling is uncontrolled when the R702W gene is mutated, protecting the organism against the harm caused by oxidative stress induced by *H. pylori (* 109). Thus, it is essential to consider how the gastric MALT lymphoma is influenced by the NOD2 gene (110) (**Table 2**).

**Table 2:** A partial list of signaling pathways linked to oxidative stress in gastric cancer.

Signaling pathways	Reference
Cell cycle regulators: Cyclin D and Cyclin E; p53, p21^Waf1/Cip1^ and p27^Kip1^	(111, 112) (113, 114)
COX-2/PGE2 and LOX/leukotrienes signaling	(115–118)
E-cadherin and Wnt/β-catenin signaling	(119, 120)
EGFR, HER2 and Ras/MAPK signaling	(121, 122)
FAK signaling	(123, 124)
Grb2/HER2 signaling	(125)
Hedgehog signaling	(126)
HIF-1α signaling pathway	(127, 128)
Hippo signaling Pathway	(129, 130)
JAK/STAT signaling	(131)
Matrix metalloproteinase and plasminogen activator system	(132, 133)
MUC1 mucin-mediated signaling pathways	(134)
NF-κB signaling	(135)
Notch signaling	(136)
PI3K/AKT/mTOR signaling	(137, 138)
PGD2/PTGDR2 signaling	(139)
STAT3 pathway	(140, 141)
TLR4 signaling	(142)
TGFβ, bone morphogenetic protein and activin signaling	(143, 144)
VEGFR‐3 signaling	(145)
WNT-β-catenin-TCF signaling pathway	(146)

## 5 Potential oxidative stress-related therapeutic targets in gastric cancer

Regulation of redox homeostasis is crucial because increasing oxidative stress has a role in all stages of carcinogenesis either initiating/stimulating tumorigenesis and promoting cancer cells transformation/proliferation or leading to cell death. Enhancing antioxidant defense capability decreases ROS as a result of one strategy (**Table 3**). However, utilizing antioxidants has been shown to change the effectiveness of treatment and, in some cases, even speed up the development of tumors.

**Table 3:** Antioxidant therapy.

Compound	Target	Reference
GSK2606414 (GlaxoSmithKline)	PERK	(147)
Statins	Autophagy	(148)
Gastrin	Autophagy	(149)
S-allyl cysteine	GSH	(150)
β-carotene	NADPH oxidase	(151)
Omega-3 fatty acids	Inflammatory and antioxidant	(152)
HsrA	Protein expression and redox homeostasis	(153, 154)
Curcumin and Res	Apoptosis-regulated genes	(155)

According to a recent study, the garlic compound S-allyl cysteine has anti-inflammatory and antioxidant properties, which greatly raises the GSH levels in the liver, gastric tissue, and serum of rat models of gastric cancer, and lowers the risk of developing gastric cancer (156). In experimental settings using AGS cells infected with *H. pylori* strains, GSH levels are lower in individuals with gastric cancer than in those with duodenal ulcers, indicating a more severe oxidative stress response to gastric cancer with *H. pylori* infection (157). The level of GSH and the ratio of GSSG/GSH significantly decline in patients of gastric cancer with *H. pylori* infection, and glutamine levels are also low. Additionally, the production of hydrogen peroxide is encouraged, aggravating the effects of oxidative stress. However, GSH therapy is proved successful in alleviating the high ROS buildup (158). In conclusion, intestinalization in the gastric host cells is caused by low GSH levels. Therefore, the risk of *H. pylori*-induced carcinogenesis of gastric mucosal may be ameliorated in rats by raising their GSH levels, which may also prevent oxidative stress damage (108).

Antioxidants, such as vitamin E and selenium, have been the subject of numerous research in this context. In 1993, the first large, randomized, double-blind, primary prevention trial to investigate the potential cancer prevention benefits of supplementing with vitamin E, selenium and β-carotene was conducted, and the cocktail has been found to dramatically lower mortality from gastric cancer (159). Interestingly, the protective effects of these antioxidants can still be noticeable ten years after the end of supplementation (160). Clinical studies have shown that consistent oral dose of β-carotene is advantageous for lowering bacterial colonization by 48% (151). It has been proposed that intake of diet rich in vitamin C, carotenoids, and alpha-lipoic acid (α-LA) may lessen the morbidity of gastric disease linked to *H. pylori* infection. α-LA, a naturally occurring dithiol with antioxidant and anti-inflammatory function, can decrease the interaction between Nrf2 and Keap1, inhibit the pro-inflammatory cytokine IL-8 production and minimize the infection *via* the Nrf2/HO-1 pathway in the AGS cells (161). It is reported that omega-3 fatty acids inhibit the oxidation of polyunsaturated long-chain fatty acids and boost the antioxidant and anti-inflammatory effects of other nutrients (162). However, omega-3 may result in oxidative stress, and the process is associated with the suppression of the production of antioxidant enzymes. Therefore, antibiotics such clarithromycin, metronidazole, quinolones, amoxicillin, and tetracycline to counteract the oxidative effects of omega-3 is recommended (74). The expression of SOD2 (Mn-SOD), superoxide anion scavenger, is elevated, but the expression of SOD1 (copper/zincSOD) is decreased while comparing gastric cancer tissues with their matching normal mucosa. In specifically, the Mn-SOD ratio (levels in normal and malignant tissue) is demonstrated as an independent predictive indicator in patients of gastric cancer, and it appears to be therapeutically relevant for the survival of patients, the higher the ratio, the poorer overall survival (163). MnSOD is elevated in primary tumors with lymph node metastases while comparing gastric cancer patients with and without metastasis, indicating that MnSOD and ROS are involved in metastasis (164).

More importantly, it is necessary to block oxidative stress completely sometimes. For instance, HsrA, the *in vivo* exclusive regulator for epsilon proteobacteria, is involved in altering redox homeostasis and protein expression. Consequently, it may serve as a potential therapeutic target to eradicate *H. pylori (* 153, 165). The increased expression of apoptosis-regulated gene in the gastric host cells of patients with *H. pylori* infection, such as BID, ZMAT3, PMAIP1 and FAS, can also be successfully controlled by the combination of curcumin and Res, which causes apoptosis to decline (166, 167).

## 6 Conclusion

Gastric cancer is the third leading cause of cancer-related death worldwide. Free radicals and oxidative stress are continuously imposed upon cells in tissues and organs on a regular basis. More and more evidences show that ROS functions an essential role in the gastric cancer. Despite a number of mechanisms have been discussed in this review, most of the ROS-induced signaling targets are yet unknown. The elevated ROS production in gastric cancer can initiate genotoxic consequences, contributing to genetic instability, DNA damage, metabolic adaptation, drug resistance and occasional cell death. However, certain amounts of ROS can be advantageous because they trigger the antioxidant defense system and shield cells. There is an urgent need to find selective and readily available therapeutic therapies for gastric cancer and gastric cancer-predisposed patients. In order to treat and prevent ROS in gastric cancer, it may be crucial to focus on the enhancement of ROS by neutralizing antioxidants to induce cancer cell death, and the inhibition of ROS activity or increase of antioxidant capacity to regulate pro-tumorigenic signaling pathways. Nevertheless, considering that multiple studies have connected some dietary antioxidants with a rise in cancer incidence, it will be crucial to thoroughly investigate all biochemical reactions within cancer cells, including their precise targets and downstream effects while boosting antioxidant capacity. More researches are needed to put on the agenda to explore the function of elevated ROS and identify the exact ROS target pathways that will be most beneficial in treating gastric cancer.

## Author contributions

All authors listed have made a substantial, direct, and intellectual contribution to the work and approved it for publication.
